# β-Cell Function and Glucose Tolerance in Persons With Multiple Islet Autoantibodies Randomized to a Gluten-free Diet

**DOI:** 10.1210/jendso/bvaf073

**Published:** 2025-05-07

**Authors:** Marlena Maziarz, Jaakko J Koskenniemi, Maria Månsson Martinez, Lampros Spiliopoulos, Falastin Salami, Jorma Toppari, Jukka Kero, Riitta Veijola, Päivi Tossavainen, Sauli Palmu, Carin Andrén Aronsson, Markus Lundgren, Henrik Borg, Anastasia Katsarou, Helena Elding Larsson, Mikael Knip, Olivia Lou, Jessica L Dunne, Carina Törn, Åke Lernmark

**Affiliations:** Department of Clinical Sciences, Lund University CRC, Skåne University Hospital, 214 28 Malmö, Sweden; Department of Pediatrics, Turku University Hospital, 20520 Turku, Finland; Research Centre for Integrative Physiology and Pharmacology, Institute of Biomedicine, and Centre for Population Health Research, University of Turku, 20014 Turku, Finland; Department of Clinical Sciences, Lund University CRC, Skåne University Hospital, 214 28 Malmö, Sweden; Department of Clinical Sciences, Lund University CRC, Skåne University Hospital, 214 28 Malmö, Sweden; Department of Clinical Sciences, Lund University CRC, Skåne University Hospital, 214 28 Malmö, Sweden; Department of Pediatrics, Turku University Hospital, 20520 Turku, Finland; Research Centre for Integrative Physiology and Pharmacology, Institute of Biomedicine, and Centre for Population Health Research, University of Turku, 20014 Turku, Finland; Department of Pediatrics, Turku University Hospital, 20520 Turku, Finland; Research Centre for Integrative Physiology and Pharmacology, Institute of Biomedicine, and Centre for Population Health Research, University of Turku, 20014 Turku, Finland; Department of Pediatrics, Research Unit of Clinical Medicine, MRC Oulu, University of Oulu, 90014 Oulu, Finland; Department of Children and Adolescents, Oulu University Hospital, 90220 Oulu, Finland; Department of Pediatrics, Research Unit of Clinical Medicine, MRC Oulu, University of Oulu, 90014 Oulu, Finland; Department of Children and Adolescents, Oulu University Hospital, 90220 Oulu, Finland; Tampere Center for Child, Adolescent and Maternal Health Research, Tampere University and Department of Pediatrics, Tampere University Hospital, 33521 Tampere, Finland; Department of Clinical Sciences, Lund University CRC, Skåne University Hospital, 214 28 Malmö, Sweden; Department of Clinical Sciences, Lund University CRC, Skåne University Hospital, 214 28 Malmö, Sweden; Department of Clinical Sciences, Lund University CRC, Skåne University Hospital, 214 28 Malmö, Sweden; Department of Clinical Sciences, Lund University CRC, Skåne University Hospital, 214 28 Malmö, Sweden; Department of Clinical Sciences, Lund University CRC, Skåne University Hospital, 214 28 Malmö, Sweden; Research Program for Clinical and Molecular Metabolism, Faculty of Medicine, University of Helsinki, 00014 Helsinki, Finland; Breakthrough T1D (Formerly JDRF International), New York, NY 10281, USA; Breakthrough T1D (Formerly JDRF International), New York, NY 10281, USA; Department of Clinical Sciences, Lund University CRC, Skåne University Hospital, 214 28 Malmö, Sweden; Department of Clinical Sciences, Lund University CRC, Skåne University Hospital, 214 28 Malmö, Sweden

**Keywords:** gluten-free diet, islet autoantibodies, oral glucose tolerance test, intravenous glucose tolerance test, continuous glucose monitoring, type 1 diabetes

## Abstract

**Purpose:**

A randomized clinical trial was conducted to evaluate the impact of a gluten-free diet (GFD) on β-cell function and glucose tolerance in persons with multiple islet autoantibodies.

**Methods:**

Individuals (n = 59; median age 11 years) with multiple islet autoantibodies were recruited to a randomized clinical trial between April 2016 and April 2021. The participants were randomized to a GFD (n = 30; female n = 14) or a normal diet (ND) (n = 29; female n = 16). The study was conducted at 6 clinical research centers in Finland and Sweden, with a dietary intervention for 17 months followed by a 6-month washout on a ND. The primary outcomes were (1) the proportion of participants going from normal glucose tolerance at the time of the randomization to abnormal glucose tolerance by 18 months, (2) a change in first-phase insulin response in IV glucose tolerance tests between randomization and 18 months, and (3) a change in C-peptide area under the curve in oral glucose tolerance test between randomization and 18 months.

**Results:**

We did not find differences between participants randomized to GFD and ND in any of the glucose tolerance outcomes. No serious adverse events or adverse events related to a GFD were noted.

**Conclusion:**

Being on a GFD was not found to differ from being on a ND in preserving β-cell function or maintaining normal glucose tolerance in persons with multiple islet autoantibodies.

The ability to predict type 1 diabetes (T1D) has accelerated the search for therapies that aim at preventing or delaying T1D [1, 2]. Strong genetic and epidemiological associations between T1D and celiac disease suggest that gluten, the main driver of celiac disease, may also affect the pathogenesis of T1D [3]. Experimental studies suggest that a gluten-free diet (GFD) may prevent or delay T1D [3, 4]. Furthermore, a GFD appeared to slow the increase in hemoglobin A1c (HbA1c) in a randomized study in patients with newly diagnosed T1D with or without concurrent celiac disease [5].

Among individuals who have 2 or more islet autoantibodies against either insulin (IAA), glutamic acid decarboxylase (GADA), islet antigen-2 (IA-2A), or zinc transporter 8, 70% to 85% progress to T1D within the next 10 to 15 years [6, 7]. Once an individual has reached the clinical onset of diabetes, β-cell function has already declined, and restoring function is difficult. This presents an opportunity to preserve β-cell function through interventions, such as a GFD. Seven children who tested positive for islet autoantibodies and were born to parents with T1D were given a GFD for 12 months. The diet did not affect their autoantibody levels, and 2 of the children developed diabetes [8]. The authors concluded no effect of GFDs and suggested that earlier implementation might be a next step [9] as also discussed in a recent review [3]. The heterogeneity and possible different phenotypes in T1D [10] would justify randomized controlled trials (RCTs) on the potential effect of GFDs on β-cell function and glucose tolerance in individuals with multiple autoantibodies. Despite the lack of evidence, GFDs have been advocated to prevent diseases, including T1D [3, 11].

To investigate this, an RCT was conducted to compare a GFD with a ND, with the primary aim to evaluate the effectiveness of a 17-month GFD intervention on β-cell function and glucose tolerance in individuals with multiple islet autoantibodies. Specifically, in this study we compare the GFD and ND arms in terms of β-cell function and glucose tolerance as measured by oral glucose tolerance test (OGTT), IV glucose tolerance test (IVGTT), and HbA1c, as well as continuous glucose monitoring. The 3 primary outcomes of the study were (1) the proportion of participants going from normal glucose tolerance (NGT) at the time of the randomization to abnormal glucose tolerance (AGT) by 18 months, (2) a change in the first-phase insulin response between randomization and 18 months based on IVGTTs, and (3) a change in C-peptide area under the curve (AUC) in OGTT between randomization and 18 months.

## Materials and Methods

### Study Design

In this open-label randomized controlled clinical trial, 59 participants were enrolled to follow either a GFD or ND (control group). The study was conducted at 6 centers: Lund University Clinical Research Center in Malmö, Kristianstad, and Helsingborg in Sweden and the Type 1 Diabetes Prediction and Prevention (DIPP) [12] study research centers in Oulu University Hospital, Turku University Hospital, and the Tampere University Hospital in Finland. All centers except Tampere are The Environmental Determinants of Diabetes in the Young (TEDDY) [13] study centers and accordingly are harmonized in blood sampling, processing, local laboratory analyses, and sample storage [14]. The study was approved by the Regional Ethical Committee in Lund as well as by the Swedish Ethical Review Authority and by the Ethics Committee of the Hospital District of Southwest Finland in Turku, Finland. Written informed consent was obtained from participants or their legal guardians for those under 18, with assent provided by the children under 18 for participation in the TEDDY Family (TEFA) study.

### Participants

Islet autoantibody-positive participants at risk of T1D were identified as described [15] and extended to the following studies for a potential inclusion in the present TEFA study: TEDDY [16], Diabetes Prediction in Skåne [17], DIPP study in Finland [18], and TrialNet [19]. The eligibility criteria were (1) age range 2 to 49.99 years and (2) history of positivity for 2 or more islet autoantibodies [GADA, IA-2A, IAA, or isoforms at position 325 for arg, tryptophan, or glutamine (ZnT8R/W/QA)] in 2 consecutive samples. The exclusion criteria were ongoing immunosuppressive therapy (topical or inhaled glucocorticoids were accepted), clinical diabetes, positivity for tissue transglutaminase autoantibodies, celiac disease, treatment with any antidiabetes medications, clinically relevant abnormal hematology results at screening, participation in any clinical drug trial within the previous 3 months of screening, presence of other serious diseases, or positivity for HLA DQB1*06:02 known to reduce the risk of T1D. Results from baseline observations prior to randomization were reported [15, 20].

### Randomization

The participants were recruited by the research nurses and coordinators and enrolled by study physicians. Our target for recruitment was 30 participants per diet arm (n = 60). This sample size was arrived at based on a simulation study [see the study protocol (Månsson Martinez M, Lernmark Å, TEFA Family Prevention: Gluten-free Diet to Preserve β-cell Function; ClinicalTrials.gov identifier: NCT02605148; updated April 4, 2025; https://clinicaltrials.gov/study/NCT02605148)] [21].

**Figure 1.**
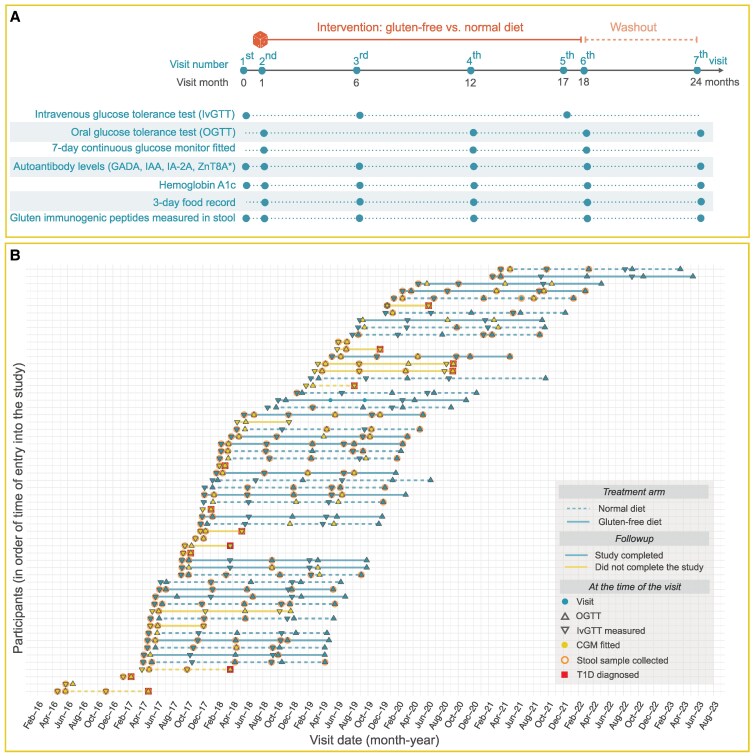
A schematic summarizing the design (A) and implementation (B) of the TEFA study conducted between 2016 and 2023. (A) The design of the TEFA study with visit numbers and the corresponding months in the study shown at the top. The time of randomization is indicated with a dice at visit 2; the intervention and the washout periods are shown at the top. The lower part of (A) shows the visits at which the different types of data were collected. The boxes highlighted in yellow indicate the part of the flow diagram from the time of randomization onwards. The main part of (B) shows the details of how the study was conducted in practice, with the legend in the top left of panel (B). Specifically, we show the follow-up of the 59 participants in the TEFA study, indicating the arm they were randomized to [solid line = gluten-free diet (n = 30), dashed line = normal diet (n = 29)]. Blue lines indicate that the participant completed the study (n = 40, 68%), yellow if not. Each visit is shown as a blue dot, with the following overlayed over the visit indicator if they occurred at that visit: gray triangle pointing up = OGTT, grey triangle pointing down = IVGTT, yellow dot = continuous glucose monitor was fitted and stayed on for 7 days after the visit, pink circle = stool sample was collected, red square = type 1 diabetes was diagnosed based on either IVGTT or OGTT (n = 13). Abbreviations: IVGTT, IV glucose tolerance test; OGGT, oral glucose tolerance test; TEFA, TEDDY Family.

After initial assessment of glucose tolerance, randomization was done after completion of the OGTT at visit 2 (Fig. 1). Randomization was done in blocks of 6 consecutively recruited participants according to 2 country-specific randomization lists. The study coordinator in Sweden and a study administrator in Finland kept the list and assigned participants to either ND or GFD. The study nurses who enrolled participants did not have access to the randomization list.

### Laboratory Procedures

All participants were given supplements including vitamin D (20 micrograms corresponding to 800 IU/day), omega-3 fatty acid (1200 mg/day), omega-6 (510 mg/day), and probiotic supplements (*Lactobacillus reuteri* DSM 17938) to reduce possible confounders. The participants attended in-person consultation visits with a dietitian at the randomization visit and at 6, 12, and 17 months after randomization. Phone consultations were conducted at 3, 9, 15, and 24 months after randomization. Baseline characteristics are summarized in Table 1. The schedule of tests and assessments included IVGTT, OGTT, a 7-day continuous glucose monitor (CGM), HbA1c, autoantibody testing, 3-day food records, and stool sampling to assess study compliance (Fig. 1A). Details on each of these procedures are described in the following sections.

**Table 1.** Baseline characteristics (at the randomization visit) of the TEDDY Family study participants randomized to either gluten-free diet or normal diet

Characteristics	Gluten-free diet	Normal diet
	(n = 30)	(n = 29)
Demographics		
Age (years)		
Median (Q2, Q4)	11.6 (8.7, 16.3)	10.5 (6.6, 15.4)
<20, n (%)	25 (83.3)	26 (89.7)
≥20, n (%)	5 (16.7)	3 (10.3)
Sex, n (%)		
Female	14 (46.7)	16 (55.2)
Male	16 (53.3)	13 (44.8)
Country, n (%)		
Finland	11 (36.7)	12 (41.4)
Sweden	19 (63.3)	17 (58.6)
Anthropometrics, median (Q2, Q4)		
Weight, kg	43.7 (29.9, 68.4)	41.6 (20.4, 55.1)
Height, cm	150 (134, 169)	150 (118, 164)
BMI z-score	0.4 (−0.3, 1.5)	0 (−0.9, 0.9)
Number of autoantibodies, n (%)		
0*^a^*	0 (0.0)	1 (3.4)
1*^a^*	4 (13.3)	4 (13.8)
2	13 (43.3)	10 (34.5)
3	11 (36.7)	11 (37.9)
4	2 (6.7)	3 (10.3)
Glucose tolerance measures, median (Q2, Q4)		
HbA1c		
%	5.2 (5.0, 5.5)	5.1 (5.0, 5.2)
mmol/mol	34 (31, 37)	32 (31, 33)
FBG, mmol/L	5.2 (4.9, 5.7)	5.3 (4.8, 5.8)
FPIR (mU/mL) based on IVGTT	94.0 (50.0, 157.5)	78.8 (37.2, 138.2)
Glucose AUC based on OGTT	970 (836, 1057)	910 (830, 1066)
C-peptide AUC based on OGTT	189 (111, 252)	183 (128, 252.7)
Glucose tolerance and T1D status at baseline based on FBG, n (%)		
NGT*^b^*	20 (66.7)	18 (60.0)
AGT*^c^*	8 (26.7)	8 (26.7)
T1D*^d^*	2 (6.7)	3 (10.0)

Q2 and Q4 refer to the 25th and the 75th percentiles.

Abbreviations: AUC, area under the curve; BMI, body mass index; FBG, fasting blood glucose; FPIR; first phase insulin release; HbA1c, hemoglobin A1c; IVGTT, IV glucose tolerance test; OGGT, oral glucose tolerance test; T1D, type 1 diabetes.

^a^Nine participants in our study who lost at least 1 autoantibody between the time of being selected as a potential candidate for our study based on their autoantibody status ascertained in 1 of the studies we recruited from and the baseline visit, at which the autoantibody status was ascertained in our study.

^b^NGT: FBG < 6.0 mmol/L; OGTT 120 minutes ≤11.1 mmol/L or HbA1c < 39 mmol/L.

^c^AGT: FBG >6.1 - < 6.9 mmol/L; OGTT at either 30, 60 or 90 minutes ≥ 11.1 mmol/L; OGTT 120 minutes ≥7.8 - < 11.1 mmol/L or HbA1c ≥ 39-<48 mmol/L.

^d^T1D: FBG >7.0 mmol/L; OGTT 120 minutes ≥ 11.1 mmol/L or HbA1c ≥ 48 mmol/L.

#### Detection of gluten immunogenic peptides in stool

Stool samples were obtained at visits 1 to 7. The samples (about 5 g) were either taken at home and brought frozen at −20 C to the clinic or obtained at the clinic visit. All stool samples were stored at −80 C until all visits had been completed in June 2023 (Fig. 1B). A quantitative ELISA sandwich assay (iVYLISA GIP Stool, Biomedal Cat# M169, RRID:AB_3661982, Biomedal, Sevilla, Spain) was used to quantify gluten immunogenic peptides (GIP) (primarily 33-mer peptides) in stool samples. All samples were assayed in duplicates according to instructions from the manufacturer. The detection limit (78 ng/g sample) was used as a threshold for positivity. The highest detection level was 1250 ng/g sample. The mean coefficient of variation for duplicate determinations was 5.1% (n = 127).

#### Analysis of islet autoantibodies

Whole blood was drawn into a syringe and transferred into a serum separation tube, allowed to clot at room temperature, and centrifuged (800×g at 4 °C for 20 minutes) to separate serum from clot. Serum was aliquoted into freezer storage tubes and stored frozen at −70 °C in cryovials. The samples were analyzed in Malmö, Sweden, for GADA, IA-2A, IAA, and ZnT8Q/W/RA; in Oulu, Finland, for GADA, IA-2A, and IAA; and in Helsinki, Finland, for ZnT8A [15, 20]. All laboratories participated in the last Islet Autoantibody Standardization Program (IASP) workshop in 2020 [22]. The IASP interlaboratory quality control comparisons report the coded workshop samples from T1D patients as percent sensitivity and from controls as percent specificity. In the IASP report from 2020, GADA in Malmö showed 64% sensitivity and 97.8% specificity, and corresponding values in the DIPP Laboratory in Oulu were 60% sensitivity and 97.8% specificity. IA-2A in Malmö achieved 82% sensitivity and 93.3% specificity and in Oulu 76% sensitivity and 100% specificity. IAA in Malmö showed 20% sensitivity and 100% specificity compared to 40% sensitivity and 96.7% specificity in Oulu. ZnT8R/W/QA (3 variants at position 325) showed 66% sensitivity and 100% specificity in Malmö, and ZnT8(R,W)A combined showed 74% sensitivity and 100% specificity in the Helsinki laboratory.

#### OGTT and IVGTT

After fasting overnight (since midnight the night before), oral glucose was administered in a dose of 1.75 g/kg body weight to a maximum of 75 g, as a solution in flavored water, consumed within 5 minutes as harmonized in the TEDDY study [14]. A 6-time point OGTT was performed with venous samples at −10, 0, 30, 60, 90, and 120 minutes, which includes sampling for glucose, insulin, and C-peptide at all time points. Glucose was measured locally.

#### HbA1c, glucose, insulin, and C-peptide

Consensus HbA1c measurements defined normal (<39 mmol/mol) (<5.7%), prediabetes (≥39-47 mmol/mol) (≥5.7-6.4%), and diabetes (≥48 mmol/mol) (≥6.5%) levels [23, 24].

The HbA1c reference ranges were 27 to 42 mmol/mol in Malmö and 20 to 42 mmol/mol in Oulu and Turku [15]. The following reference values were, as previously published [15], in Malmö, p-glucose 1 month-18 years: 3.3 to 5.6 mmol/L and above 18 years: 4.2 to 6.3 mmol/L, s-insulin (Roche Cat# 12017547, RRID:AB_2756877): < 25 mIE/L, s-C-peptide (Roche Cat# 07 027 168 190, RRID:AB_2893132): 0.37 to 1.5 nmol/L; in Oulu, p-glucose 4.2 to 6.0 mmol/L, s-insulin (Roche Cat# 12017547, RRID:AB_2756877): 5 to 20 mU/L, s-C-peptide (Roche Cat# 07 027 168 190, RRID:AB_2893132): > 0.9 nmol/L; and in Turku, p-glucose 4 to 6 mmol/L, s-insulin (Roche Cat# 12017547, RRID:AB_2756877): 2.6 to 25 mU/L, s-C-peptide (Roche Cat# 07 027 168 190, RRID:AB_2893132): 0.37 to 1.47 nmol/L.

#### Continuous glucose monitoring

Dexcom sensors (Dexcom, Inc., San Diego, CA) were G4 initially and later G6 in Finland, while G5 was used in Sweden to collect interstitial glucose level data every 5 minutes for 7 days. The G4 and G5 instruments were calibrated twice a day with finger-prick glucose; G6 does not require external calibration. The data were summarized for each participant for each 7-day period using 9 CGM metrics: median; interquartile range; range; the proportion of time spent above 120 mg/dL (6.7 mmol/L, TA120), above 140 mg/dL (7.8 mmol/L, TA140) [25], and above 160 mg/dL (8.9 mmol/L, TA160); continuous overall net glycemic action (CONGA) [26]; mean amplitude of glucose excursions (MAGE) [27]; and mean of daily differences (MODD) [27].

#### Dietary assessments

At baseline (visit 1, month 0), participants were instructed to document all foods and beverages consumed over a 3-day period (2 weekdays and 1 weekend day) within 10 days before the scheduled visit. The 3-day food records were collected at visits 2, 3, 4, 6, and 7. Dietary intake in 3-day food records was analyzed using separate national food composition databases from both countries. The total energy intake (kcal/day) and the total intake of carbohydrates, protein, and fat as well as the proportion of energy from protein, fat, and carbohydrates were estimated. We also compared the intake of 8 nutrients that have previously been shown to differ between a GFD and ND [28].

The intake of each food and ensuing food groups were recorded in a food database with a connected software as described [29]. The participants recorded recipes of each dish, and the software broke them down by their ingredients. The Swedish National Food Composition Database [Swedish National Food Agency, Uppsala, Sweden (https://www.livsmedelsverket.se/en/food-and-content/naringsamnen/livsmedelsdatabasen; accessed April 28, 2023)] and the Finnish Fineli (https://fineli.fi/fineli/en/index) Food Composition Database (Finnish Institute for Health and Welfare), respectively, were used to calculate the nutrient content and standard recipes of food such as bread, sweet bakery, pancakes, pizza, etc. If the recorded recipes were not available in the databases, new recipes were created and added to the local databases. Household measurements, food models, pictures [based on the Swedish National Food Agency (*Livsmedelsverket*)], and shapes of foods as references were used to estimate portion sizes. The participants used a detailed information folder with examples on how to indicate mealtime, location, adequate descriptions of foods and beverages, and quantity of intake. To provide quality control of the 3-day food records, the dieticians and the study nurses in both countries reviewed the records with the participants at each visit. Each participant in the GFD group was asked about compliance with the diet between and during the visits.

### Outcomes

The 3 primary outcomes of the study were (1) the proportion of participants going from NGT at the time of the randomization to AGT by 18 months, (2) a change in the first-phase insulin response between randomization and 18 months based on IVGTT, and (3) a change in C-peptide AUC in OGTT between randomization and 18 months. A participant was said to have AGT if any of the following were true: (1) fasting plasma glucose ≥6.1 < 11.1 mmol/L; (2) maximum plasma glucose at 30, 60, and 90 minutes ≥ 11.1 mmol/L in the OGTT; (3) 120 minutes plasma glucose ≥7.8 mmol/L in OGTT; or (4) HbA1c ≥ 39 mmol/mol (5.7%). This definition is consistent with other definitions for AGT [6] and the American Diabetes Association/World Health Organization definition of impaired glucose tolerance and impaired fasting glucose [30], with 1 difference being that we used 6.1 mmol/L instead of 5.6 mmol/L as the threshold for fasting plasma glucose [30]. First phase insulin release (FPIR) was calculated as the sum of plasma insulin at 1 and 3 minutes after administering the IV glucose bolus, as described [15].

As part of an exploratory analysis, we considered the following secondary outcomes: changes from baseline in glucose AUC, fasting C-peptide (both based on OGTT), HbA1c, and Homeostatic Model Assessment version 2 (HOMA2)-insulin resistance (IR), -β-cell function index, and -insulin sensitivity index and 9 summary statistics for summarizing 7 days of glucose measurements from a CGM. Data availability (ie, nonmissingness) for key measures ranged from 30% (for CGM) to 99% (for fasting plasma glucose) (Table 2).

**Table 2.** Number of available observations compared to theoretical maximum according to the study protocol

Measures	Visits	Gluten-free diet	Normal diet
		(% nonmissing)	(% nonmissing)
Anthropometry	1-7	100	100
OGTT (C-peptide at 0'−120')	2, 4, 6, 7	91-94	96-97
OGTT (AUC c-peptide 0'−120')	2, 4, 6, 7	94	98
OGTT (glucose at 0'−120')	2, 4, 6, 7	97-99	98-99
OGTT (insulin at 0'−120')	2, 4, 6, 7	92-94	96-98
Fasting glucose (IVGTT or OGTT)	1-7	99	99
Fasting insulin (IVGTT or OGTT)	1-7	88	86
IVGTT (FPIR, fasting glucose, and insulin)	1, 3, 5	92-95	94-99
IVGTT K-value*^a^*	1, 3, 5	88	97
CGM*^b^*	2, 4, 6	48	30
HbA1c	1-7	97	97
HOMA2 indices	1-7	88	86
3-day food records	2, 3, 4, 6, 7	89	89
GADA, IAA, IA-2A	1-7	100	97
ZnT8A	1-7	96	92
ZnT8R/W/QA	1-7	70	55
Stool GIP	1-7	59	46
Stool GIP (during GFD)	2-6	71	67

Abbreviations: ', minutes; AUC, area under the curve; CGM, continuous glucose monitoring; FPIR, first phase insulin release; GADA, glutamic acid decarboxylase 65 autoantibodies; GFD, gluten-free diet; GIP, gluten immunogenic peptide; HbA1c, hemoglobin A1c; HOMA2, Homeostatic Model Assessment 2; IA-2A, islet antigen-2 antibodies; IAA, insulin antibodies; IVGTT, IV glucose tolerance test; OGGT, oral glucose tolerance test; ZnT8A, zinc transporter 8 autoantibodies; ZnT8R/W/QA, isoforms at position 325 for arginine, tryptophan, or glutamine.

^a^The proportions shown are based on Swedish participants only. No K-value data was available for the Finnish participants due to restrictions in blood sampling in Finland.

^b^The apparently low compliance was due to a computer breakdown causing loss of data.

### Adverse Events

The participants were interviewed at every visit for any occurrence of adverse events (AEs) and serious AEs.

### Statistical Analysis

Derived variables were calculated, specifically, the area under the receiver operating characteristics curve based on OGTT for glucose (0-120 and 30-90 minutes) and C-peptide AUC (0-120 and 30-90 minutes). The HOMA2 indices (IR, β-cell function index, insulin sensitivity index) were calculated using the Excel HOMA2 calculator implementation from Oxford University (https://www.rdm.ox.ac.uk/about/our-clinical-facilities-and-mrc-units/DTU/software/homa/download; accessed July 5, 2023), ensuring that the fasting glucose and insulin were converted to correct units before calculating HOMA2 indices [31]. Summary statistics from the CGM data were calculated: median, interquartile range, range, TA120, TA140, TA160, MODD, CONGA, and MAGE, with the last 3 using an R package iglu. Right-skewed variables were log2 transformed before analysis.

The AGT status was assessed based on several metrics from OGTT; specifically, an individual was defined to have AGT if any of the following were true: (1) fasting plasma glucose ≥ 6.1 < 11.1 mmol/L; (2) maximum plasma glucose at 30, 60, 90 minutes ≥ 11.1 mmol/L in the OGTT; (3) 120 minutes plasma glucose ≥ 7.8 mmol/L in OGTT; or (4) HbA1c ≥ 39 mmol/mol (5.7%). We also defined a combined outcome from AGT and T1D, where a participant was said to be AGT or T1D if they had AGT or were diagnosed with T1D at any time point during the intervention.

We compared the trajectories of each of the primary outcomes over time and compared the 2 treatment arms at each visit. We performed the same analysis for what we considered to be the secondary, or exploratory, outcomes (fasting glucose; glucose AUC 0-120 minutes; glucose AUC 30-90 minutes; HbA1c; fasting C-peptide; C-peptide AUC 30-60 minutes; HOMA2-IR, -β-cell function index, and -insulin sensitivity index).

To compare the change in the levels of the primary outcomes between the last prerandomization (pre) visit and the last visit before washout (post) between the 2 diet groups, we calculated the difference between those 2 time points (post-pre) and compared them using the Wilcoxon rank test for each outcome separately. The first prerandomization visit was visit 2 for most outcomes and visit 1 for FPIR, and the last visit before washout was visit 6 for most outcomes and visit 5 for FPIR. We performed the same analysis for the secondary outcomes. Similar comparisons were done for nutrients, as well as for GIP.

In an exploratory analysis, we compared the AGT and T1D-free, as well as T1D-free, survival functions between the 2 treatment groups over the course of the study; we used the Kaplan-Meier estimator and estimated the *P*-value for the difference between the survival curves using the log-rank test.

Unless otherwise stated, the *P*-values reported are based on the Wilcoxon test when comparing continuous variables and Fisher's exact test for comparing categorical variables. No adjustment for multiple comparisons was made; thus all *P*-values shown are nominal. Missing data was not imputed.

All analyses were performed in R v4.3.2 (r-project.org). In addition to the standard packages (such as ggplot2, here, rio, survival, survminer, tidyverse), we used an R package iglu to obtain the MODD, CONGA, and MAGE summary statistics from the CGM data.

## Results

**Figure 2.**
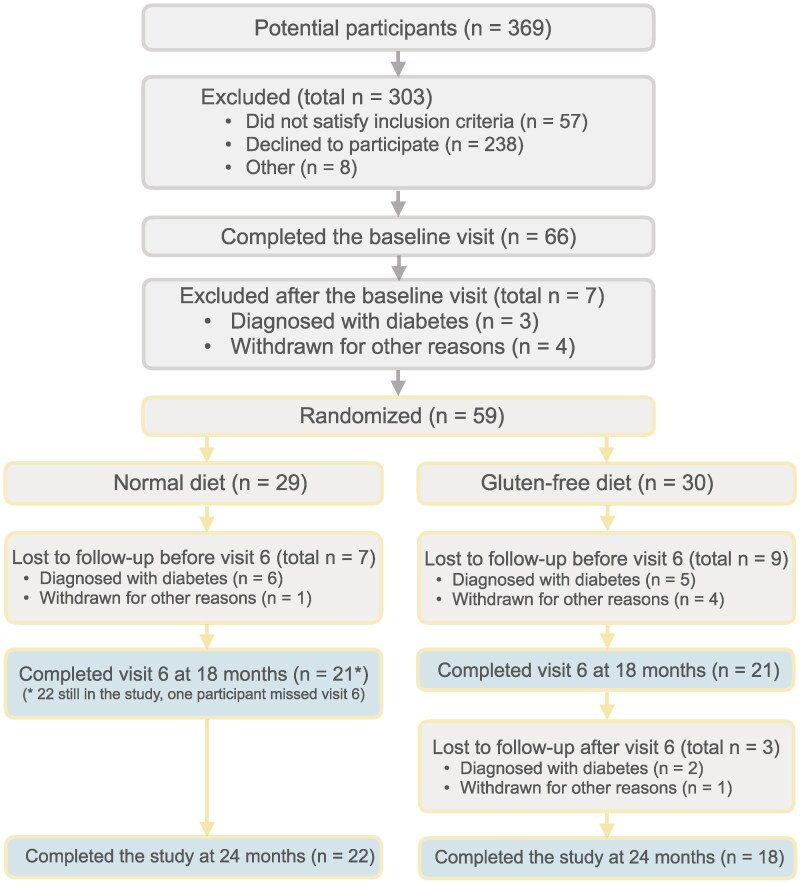
The CONSORT flow diagram of the TEDDY Family (TEFA) study.

The recruitment took place between April 2016 and April 2021, and the follow-up was completed in May 2023. A total of 66 (17.9%) multiple islet autoantibody-positive individuals 66 (17.9%) agreed to participate and to complete the baseline assessment at visits 1 and 2 (Figs. 1 and 2). Three individuals were diagnosed with T1D at either of the 2 first visits, 4 withdrew, and the remaining 59 (16.3%) were randomized to either a GFD (n = 30) or ND (n = 29). The last study visit was completed in May 2023 (Fig. 1B). The diagnosis of T1D was based on hyperglycemic values in 2 consecutive OGTTs. Therefore, some participants with AGT at randomization progressed to T1D very early in the trial.

GFD and ND participants were similar at baseline in terms of demographics, β-cell function, and characteristics of glucose tolerance (Table 1). During the 17-month intervention, 6 participants on a ND and 7 on a GFD were diagnosed with T1D (Figs. 1B and 2). Of these, 4 participants were diagnosed with T1D shortly after randomization (1 randomized to a GFD and 3 to a ND; Figs. 1B and 2).

**Figure 3.**
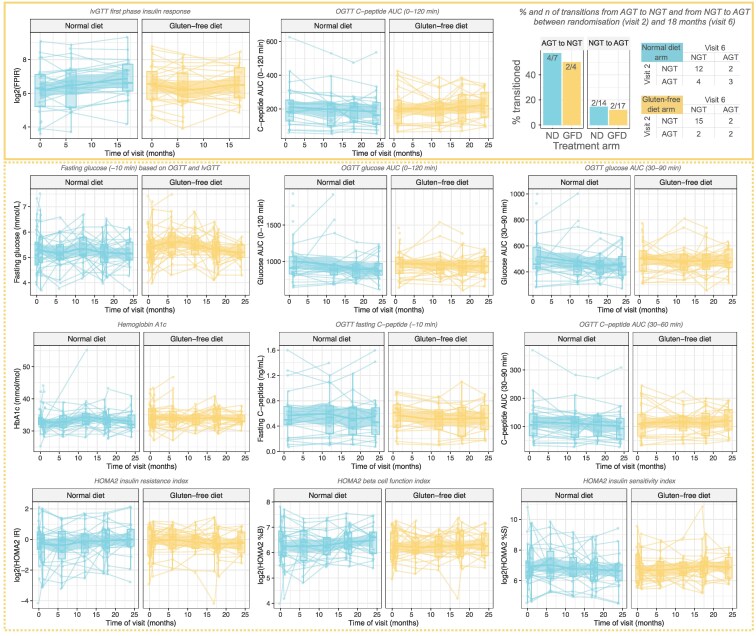
Individual trajectories over time for primary and secondary outcomes stratified by the treatment arms of normal diet and gluten-free diet, respectively. The top row shows the 3 primary outcomes (inside the solid yellow box); the secondary outcomes are shown in the lower three rows (inside the dotted yellow box). For each outcome, we show the individual trajectories over time, boxplots summarizing the distribution of the data at a given visit, and a smooth line with a 95% pointwise confidence interval, all stratified by the treatment arm (normal diet vs gluten-free diet). The type of the outcome is indicated in the title and on the y-axis, with the time in study (in months) shown on the x-axis for all except 1 plot in the top right. The primary outcomes were log2-transformed first phase insulin response based on the IVGTT; fasting blood glucose based on both IVGTT and the OGTT at −10 minutes blood sample; and the percentage and the number of transitions from AGT to NGT and vice versa over the course of the study from randomization to 18 months in each treatment arm. The secondary outcomes include glucose AUC (0-120 min and 30-90 minutes) based on OGTT, hemoglobin A1c, fasting C-peptide, C-peptide AUC (0-120 minutes and 30-90 minutes) all based on OGTT, and the HOMA2 IR, β-cell function, and insulin sensitivity indices. *P*-values for the comparisons between the treatment arms in the differences for each outcome between visit 6 compared to visit 2 (or 5 compared to 1) were not statistically significant for any of the outcomes. Abbreviations: AUC, area under the curve; IVGTT, IV glucose tolerance test; OGGT, oral glucose tolerance test.

**Figure 4.**
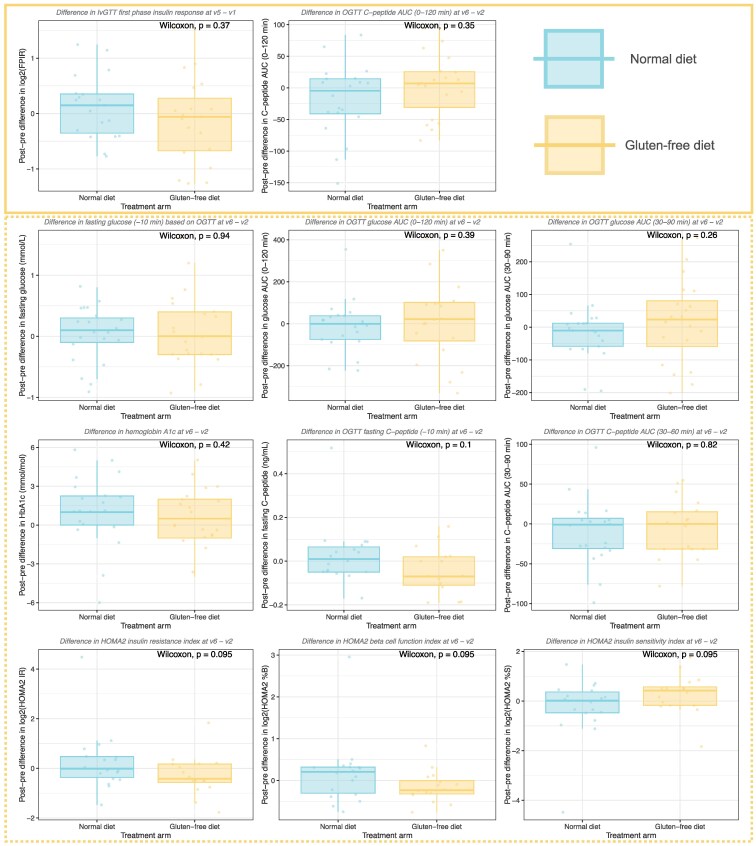
Comparison of changes over time between the treatment arms. This is a companion figure to Fig. 3 in which a comparison is made between the post-pre differences in each of the outcomes. Specifically, the “post” is the level at the last visit on treatment (visit 5 for FPIR, visit 6 for all others), and “pre” is the first visit (visit 1 for FPIR, visit 2 for all others) (y-axis). No statistically significant differences between the treatment arms were found in the change over time in any of the outcomes. Abbreviation: FPIR, first phase insulin release.

The levels of the primary outcomes, C-peptide AUC (from OGTT) and FPIR (from IVGTT), did not differ between the GFD and ND groups throughout the study, that is, between the baseline visit and any postrandomization visits (top row in Figs. 3 and 4). The third primary outcome, the proportion of participants transitioning from NGT to AGT over the course of the intervention (visit 2 to visit 6), was also not found to differ between the treatment arms (top right in Fig. 3). Comparing the 2 treatment arms in terms of the cumulative proportion of participants with AGT or T1D at any time point during the study, again, we did not find any differences, with 23/29 participants with AGT or T1D in the ND arm and 20/30 in the GFD arm.

**Figure 5.**
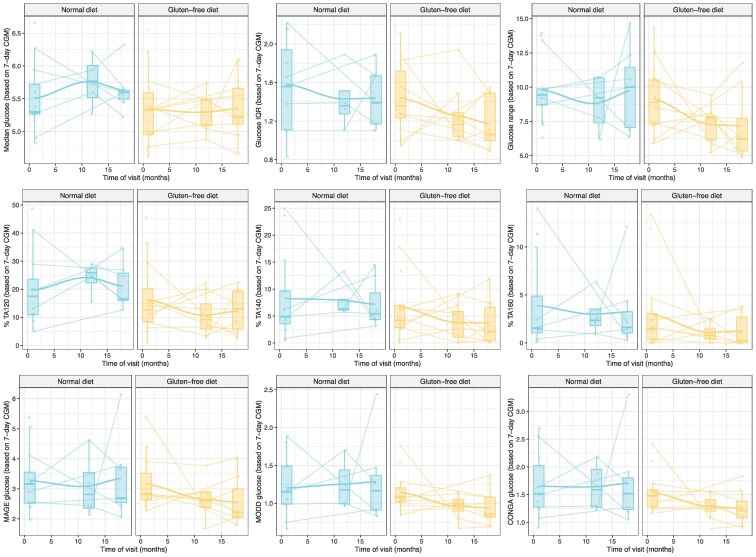
Summary of the CGM data at visits 2, 4, and 6 summarized using 9 CGM metrics and stratified by the treatment arm. Abbreviation: CGM, continuous glucose monitor.

We did not observe any differences in changes from baseline to any other time point in any of the secondary outcomes: fasting plasma glucose, fasting insulin, or homeostasis modeling metrics (HOMA-IR, -β-cell function index, and -insulin sensitivity index), which describe β-cell function and insulin sensitivity in steady state, respectively) (lower 3 rows in Figs. 3 and 4). Trajectories of CGM metrics summarizing the CGM glucose levels and variability (median glucose, glucose interquartile range, glucose range, TA120, TA140, TA160, MAGE, MODD, and CONGA) were also not found to differ between GFD and ND (Fig. 5).

**Figure 6.**
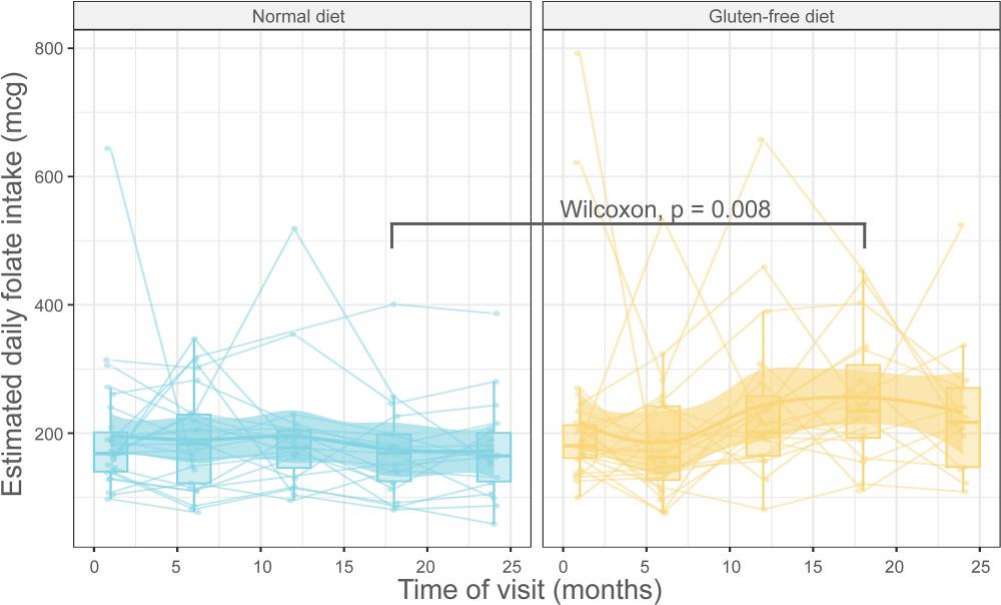
The estimated folate intake over the course of the study, stratified by the treatment arm [normal diet compared to gluten-free diet (A)]. The data in (B) compare the change in the folate intake levels between visits 2 and 6 on the normal and the gluten-free diet. The differences are shown on the y-axis and are post-pre (folate level at visit 6 minus that at visit 2). The Wilcoxon *P*-value comparing the post-pre folate intake is shown on the plot.

When comparing the intake of 8 nutrients that are commonly lower in GFDs, we noted higher levels of folate on a GFD compared to the ND at the end of the intervention period (visit 6) (*P* = .01, Fig. 6). We did not find any statistically significant differences in the intake of calcium, fiber, vitamin D, vitamin B12, magnesium, zinc, or saturated fatty acids (data not shown).

**Figure 7.**
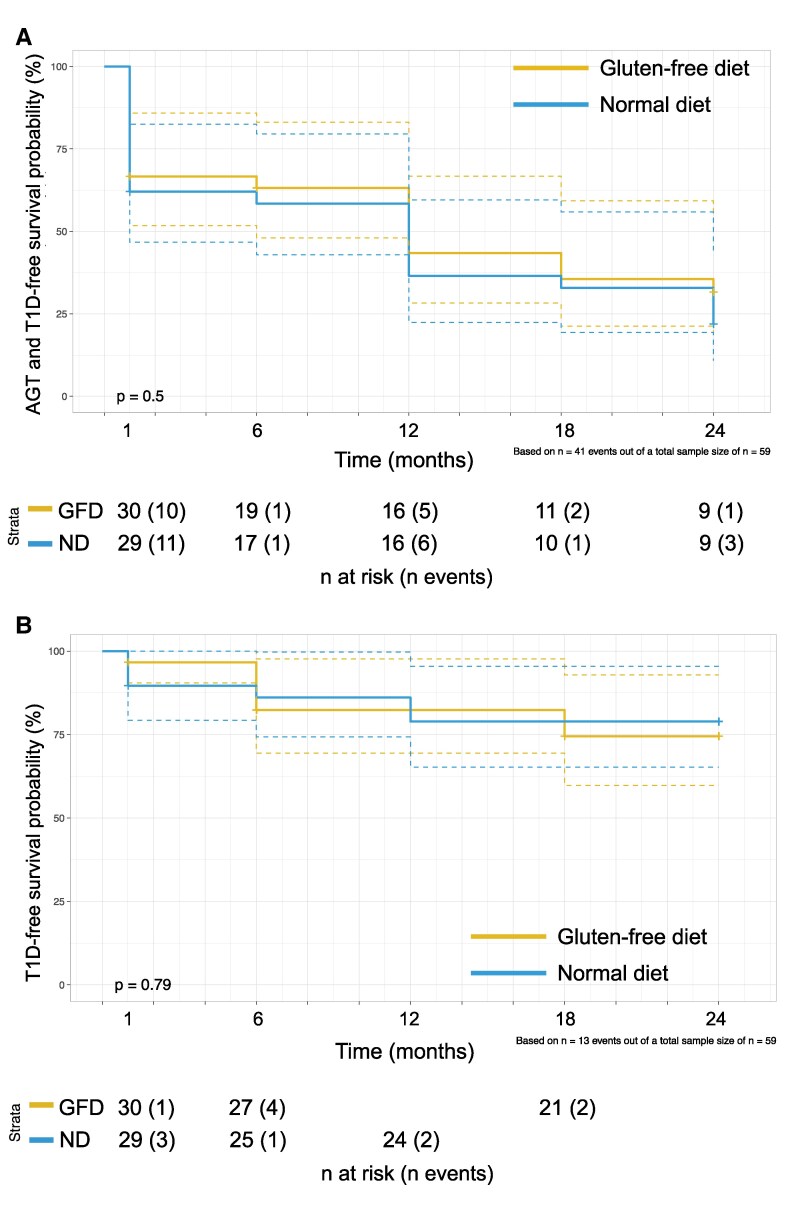
Abnormal glucose tolerance and type 1 diabetes-free survival probability (A) and type 1 diabetes-free survival probability (B) estimated using the Kaplan-Meier estimator and stratified by the gluten-free diet or a normal diet. Below each plot we report the number of participants at risk at each time point and the number of events at that time point stratified by the treatment arm.

In an exploratory analysis, we compared the 2 treatment arms in terms of rates of progression to AGT or T1D, as well rates of progression to T1D. We did not find any difference in rates of progression for either of these (Fig. 7).

**Figure 8.**
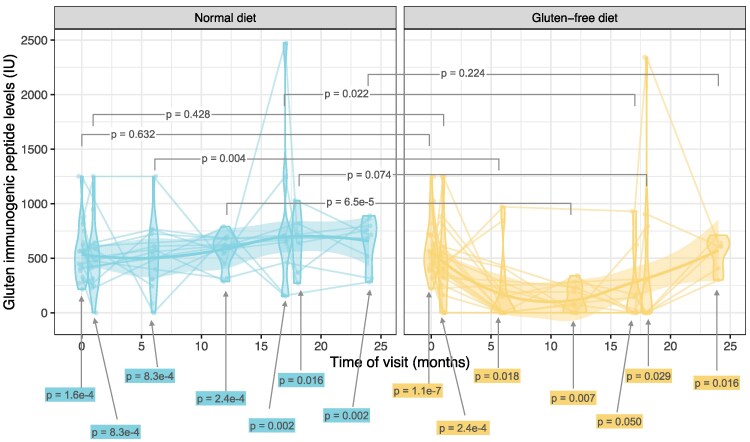
Levels of the GIP measured in stool samples at each visit, stratified by the treatment arm (ND vs GFD) and visit. The *P*-values comparing the levels of GIP between the ND and the GFD are based on a Wilcoxon 2-sample test with the null hypothesis of no difference between the 2 groups at a given visit. The *P*-values shown below the figure are based on a Wilcoxon 1-sided test with the null hypothesis of the GIP levels not being different from 0. The sample sizes for the GIP comparisons at visits 1 to 7 in the ND group were n = 17, 14, 14, 12, 9, 6, 9, respectively, and in the GFD group they were n = 23, 21, 16, 12, 8, 12, 6, respectively. Abbreviations: GFD, gluten-free diet; GIP, gluten immunogenic peptide; ND, normal diet.

Adherence to the GFD was inconsistent, as evaluated by GIP in stool sampled at visits 3, 4, and 6 (Fig. 8). In Sweden 6/16 (38%) and in Finland 8/12 (67%) of the participants were GIP-positive on a GFD compared to 7/9 (78%) and 12/12 (100%), respectively, on a ND. GIP levels in the GFD arm gradually declined, indicating a delayed elimination of gluten. After the 6-month wash-out between visits 6 and 7, GIP was positive in all participants providing a stool sample (Fig. 8).

Frequencies of AEs and their distribution between a GFD and ND showed that no serious AEs were reported, and none of the AEs were considered causally linked to a GFD. Occurrence of AEs with International Classification of Diseases, Tenth Revision (ICD-10) codes belonging to the ICD-10 chapter “Infectious and Parasitic Infections” (A00-B99) appeared less common in the ND (2/30, 7%) compared to a GFD (10/29, 35%; *P* = .03) during the intervention, whereas there were no differences between a GFD and ND in any other ICD-10 chapters (data not shown).

## Discussion

In this RCT, we did not note any differences in the primary outcomes or any of the other measures of glucose tolerance we considered between the GFD and ND arms among the RCT participants. The participants were all positive for multiple islet autoantibodies at the start of the study. However, 57/59 of the research participants had heterogeneous glucose tolerance and β-cell function at baseline [15]. Also, among 46 single autoantibody-positive individuals evaluated as part of the present protocol, all had normal responses in IVGTT and OGTT [20]. Among the enrolled participants, 69% developed T1D when they had been on a GFD or ND for less than 6 months. Finally, stool GIP was reduced during the study, but some participants had GIP toward the end of the 17-month study. These principal findings do not suggest that a GFD is superior to a ND to maintain NGT.

A strength of the present study was the prospectively planned randomized design and frequent guidance by a trained dietician as well as the GIP analyses. In addition, all participants were given vitamin D, omega-3 fatty acids, omega-6, and probiotic supplements to reduce possible confounder effects. The dieticians, trained in the TEDDY harmonized protocol [32], collected 3-day food records to verify compliance and that the participants on the GFD were not inadvertently exposed to products containing gluten. As expected, the fiber intake between the GFDs and NDs was potentially reflected in a different folate intake detectable at visit 6. These strengths, combined with the comprehensive metabolic analysis including 3-time IVGTT and OGTT (Fig. 1) as well as CGM (Fig. 7), would have seem to suffice to uncover if a GFD affects β-cell function or glucose tolerance in persons with multiple islet autoantibodies. The increased interest in CGM as outcome measures in trials as a replacement test for IVGTT and OGTT [33] lead us to include CGM in the protocol. However, the CGM measurement suffered from a loss of recorded data in some participants due to a misunderstanding on how to download the information from study participants. CGMs were well tolerated and may be considered a potential end-point measure in future studies [15, 20].

A weakness of the study was that 5 participants progressed to T1D early in the study, and 6 participants lost autoantibody multipositivity during the follow-up. As islet autoantibodies tend to disappear prior to T1D diagnosis [34, 35], this may represent a common problem when individuals with multiple islet autoantibodies are recruited [15, 36]. Three individuals lost the autoantibodies transiently (all on a GFD); another 3 lost the autoantibodies persistently (all on a ND) and therefore had only 1 autoantibody at visit 7. This reduction in the number of participants made it difficult to detect a difference between a GFD and ND in the loss of β-cell function. However, a larger sample size may not have altered the results, since we did not observe any improvement in β-cell function or glucose tolerance on the GFD compared to the ND. The expected change in folate levels was observed, indicating the efficacy of a GFD. It is unclear whether the absence of observed differences between the groups in the outcomes considered reflects a true lack of relevant differences, insufficient statistical power, or the possibility that the study timeframe was too short. However, the confidence intervals can be used to gain a sense of statistical sensitivity in our study. Several of our primary and secondary outcomes are relatively wide, meaning our study most likely lacked the power to detect very small effect sizes. As a rough guideline, our study had approximately 50% power to detect effect sizes equal to about half the width of the confidence interval for the association between a given outcome and diet group.

After the approval of the present protocol, a consensus report proposed glucose tolerance as a tool for staging the disease process among multiple autoantibody-positive individuals from stage 1 (NGT) through stage 2 (AGT) to stage 3 (hyperglycemia to meet the American Diabetes Association/World Health Organization diagnostic criteria for T1D) [6]. In the present study at visit 1 and visit 2 prior to randomization, participants positive for 3 or more autoantibodies had a lower FPIR as compared to participants with 2 autoantibodies, supporting the view that their β-cell function had deteriorated [20]. Further analysis of our 57/59 participants revealed that the sole use of 2 or more islet autoantibodies as an inclusion criterion in prevention trials is unsatisfactory and that heterogeneity in β-cell function and glucose tolerance needs to be taken into account [15]. The advances in immunotherapy [2] have fueled local screening initiatives and discussions about universal screening for islet autoantibodies [37], resulting in recent consensus guidelines [38, 39].

In conclusion, we did not find evidence to recommend a GFD to individuals with multiple islet autoantibodies in order to preserve β-cell function or maintain NGT. We interpret our results to suggest that 18 months of a GFD is unlikely to prevent the progression from NGT to AGT among individuals with multiple islet autoantibodies. Future investigations will have to carefully delineate the risk that a research participant may be close to transitioning from deteriorating glucose tolerance and β-cell function to clinical onset of diabetes. Currently, it has been possible to delay the clinical onset in islet autoantibody-positive individuals by either teplizumab [2] or physical activity [40], each for about 2 years. Our observations highlight the challenge of RCTs in multiple islet autoantibody-positive individuals aimed to prevent or delay progression to clinical onset of T1D.

## Data Availability

All datasets generated during and/or analyzed during the current study are not publicly available but are available on reasonable request. Requests for deidentified participant-level data, data dictionary and statistical code should be made to Å.L., M.M., and J.J.K. with a description of the analytical plan and purpose of the request. Study records will be stored for 10 years after completion of the study before being destroyed.

## Clinical Trial Information

This study was registered with ClinicalTrials.gov number NCT02605148.

## Abbreviations

AE: adverse event
AGT: abnormal glucose tolerance
AUC: area under the curve
CGM: continuous glucose monitor
CONGA: continuous overall net glycemic action
DIPP: Type 1 Diabetes Prediction and Prevention
FPIR: first phase insulin release
GADA: glutamic acid decarboxylase 65 autoantibodies
GFD: gluten-free diet
GIP: gluten immunogenic peptide
HOMA2: Homeostatic Model Assessment version 2
IA-2A: islet antigen-2 autoantibodies
IAA: insulin autoantibodies
IASP: Islet Autoantibody Standardization Program
ICD: International Classification of Diseases
IR: insulin resistance
IVGTT: IV glucose tolerance test
MAGE: mean amplitude of glucose excursions
MODD: mean of daily differences
ND: normal diet
NGT: normal glucose tolerance
OGTT: oral glucose tolerance test
RCT: randomized controlled trial
T1D: type 1 diabetes
TEDDY: The Environmental Determinants of Diabetes in the Young
TEFA: TEDDY Family
ZnT8A: zinc transporter 8 autoantibodies
ZnT8R/W/QA: isoforms at position 325 for arginine, tryptophan, or glutamine
